# Microbioreactor design for eukaryotic cell-based biomedical applications

**DOI:** 10.3389/fbioe.2026.1854988

**Published:** 2026-07-13

**Authors:** Gustavo Rosero, Camilo Pérez-Sosa, Julio Patricio Peñaherrera, Ana Belen Peñaherrera-Pazmiño

**Affiliations:** 1 Universidad UTE, Facultad de Ciencias de la Ingeniería e Industrias, Ingeniería Civil, Mecánica Computacional e Inteligencia Artificial Aplicada (MCIAA), Quito, Ecuador; 2 Centro de Investigación Biomédica (CENBIO), Universidad UTE, Facultad de Ciencias de La Salud Eugenio Espejo, Quito, Ecuador; 3 Facultad de Ingenierías Aplicadas y Desarrollo Industrial, Universidad Internacional Del Ecuador UIDE, Quito, Ecuador

**Keywords:** CAR T-cells, hiPSCs, microbioreactor, microenvironment, organoid, perfusion, personalized medicine, SDG 3

## Abstract

The eukaryotic cell-based biomedicine landscape is undergoing a paradigm shift, driven by the transition from static culture to highly controlled microbioreactor environments. This mini-review assembles high-value applications, including autologous cell therapies, recombinant protein production, induced pluripotent stem cell expansion, and organoid engineering. Technologically, we highlight how the integration of microfluidics into Chimeric antigen receptor T-cell manufacturing achieves numerical optimization of cell count and phenotypic distribution through automated, closed-loop workflows. Regarding bioproduction, perfusion-based microbioreactors demonstrate a six-fold increase in productivity over traditional T-flasks, utilizing at-line monitoring and advanced cell-retention configurations to minimize shear-induced damage. Beyond production, these systems are active regulators of oxygenation and tissue morphogenesis in the context of human induced pluripotent stem cells while enabling controlled 3D tissue organization. In this connection, microfluidic platforms influence extracellular matrix remodeling and developmental signaling by modulating convective flow and mechanotransduction pathways. Furthermore, automation enhances reproducibility in feeding schedules and differentiation timing. Critical to this endeavor, the ability to generate precise spatiotemporal morphogen gradients enables reproducible lineage commitment and the maturation of complex tissues through synchronized mechanical and electrical stimuli for hosting organoid growth. The microbioreactor’s superior mass transfer and low shear stress produced organoids 140% larger than static controls. While automation significantly reduces reagent consumption and inter-batch variability, challenges in scalability and material selection remain. Finally, parameters that push the microscale control boundaries are presented. Collectively, these advancements streamline therapeutic pipelines, support the replacement of animal-based testing, and align with Sustainable Development Goal 3 by accelerating the move toward personalized medicine and ethical drug screening.

## 1 Introduction

The bioreactor, originally a workhorse of the fermentation industry, has undergone a radical transformation. It has evolved from a simple vessel for microbial growth into a sophisticated, multidisciplinary system designed to recapitulate the complex environments found within the human body. This evolution supports a critical tissue engineering paradigm about expanding patient cells *ex vivo* and seeding them into 3D biomaterial scaffolds, where the bioreactor provides the precise cues needed for the resulting tissues to achieve functional competence (Radisic, 2020).

Microfluidics is defined as the science and technology of systems that process or manipulate small (10^−9^ to 10^−18^ L) amounts of fluids, using channels with dimensions of tens to hundreds of micrometres (Whitesides, 2006; Sackmann et al., 2014). This technology provides physics-based advantages, such as laminar flow and rapid diffusion, which are distinct from macroscale phenomena. As microchannels are at the same scale as cells, and cells are true tissue engineers (Radisic, 2020), microfluidic platforms provide precise spatiotemporal governance over the cellular niche. By maintaining a homogeneous flow field, consistent fluidic stimuli, and efficient mass transport, these systems ensure both experimental reproducibility and highly predictable biological outcomes, providing the controlled conditions necessary for high-yield metabolite production and the engineering of complex, cell-derived organoids (Chen et al., 2025; Aranda Hernandez et al., 2022), Current tools often force a trade-off between high throughput and dynamic control. However, the scaled-down microfluidic bioreactors referred to as microbioreactors (Hegab et al., 2013; Frey and Krull, 2020) offer a way to achieve both, streamlining the transition from initial bioprocess design to full-scale manufacturing (Totlani et al., 2023). Indeed, microbioreactors merge the compact scale of microtiter plates with the advanced tracking and regulation capabilities of larger bench-scale setups; consequently, sensor-integrated microbioreactors have emerged as highly efficient tools (Hegab et al., 2013). They represent a compelling approach for the high-throughput screening of experimental parameters (Prado and Borges, 2018).

At present, microbioreactors have fulfilled remarkable achievements. For instance, sufficient nutrient supply, dynamic fluidic stimuli and cellular behavior manipulation in practical applications (Chen et al., 2025).

**FIGURE 1:**
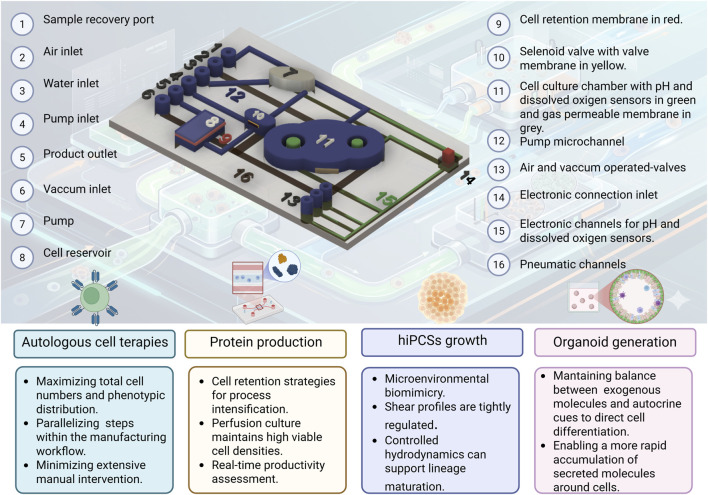
Microbioreactor main components and biomedical applications. The upper part shows the ideal microbioreactor system scheme. According to application, a microbioreactor should integrate microfluidic pumps that perfuse medium, a pneumatic line that controls valves enabled by air inlet and vacuum inlet, membranes that open and close valves, cell retention membrane, gas permeable membrane for cell oxygenation (red), electric circuits for sensors, cell reservoir, cell culture chambers provided with pH, dissolved oxygen and optical density sensors (green), sample recovery port for quality monitoring and product outlet. The lower part shows a brief summary of main biomedical applications assisted by microbioreactors. Created in BioRender and Fusion360 (Autodesk).

In this mini-review, we showcase the current landscape of microbioreactors and their pivotal role in eukaryotic cell-based biomedical applications. We specifically examine their utility in autologous cell therapies, recombinant protein production, human induced pluripotent stem cells (hiPSC) expansion and organoid engineering (Figure 1). These advances support the replacement of animal-based testing and are aligned to Sustainable Development Goal 3 (SDG 3) as they contribute to personalized and accessible medical treatments and revolutionize disease modeling, developmental biology and drug screening.

## 2 Translating laboratory bioprocesses into commercial-scale operations

### 2.1 Autologous cell therapies

Chimeric antigen receptor (CAR) T-cell therapy is a type of adoptive T-cell therapy that has shown success in treating systemic cancers. Despite ongoing advances in the immuno-oncology field, *ex vivo* manufacturing of T-cells on a commercial scale with high reproducibility and robustness remains a challenge (Amini et al., 2020). The success of T-cell expansion is measured not only by the production of a high number of cells but also by the phenotypic distribution of the resulting cell population. Therefore, Amini and collaborators (2020) (Amini et al., 2020) utilized a 24-well microbioreactor (Wiegmann et al., 2020) and a factorial experimental design in which parameters such as pH, shaking speed, and dissolved oxygen (DO) were varied over two levels: high or low. In terms of viable cell concentration (VCC), DO was determined to have no significant effect and was thus eliminated from the model. Between the two remaining factors, shaking speed had a stronger effect on the VCC model than pH. Whereas in terms of the percentage of CD8^+^ T_CM_ cells, the analysis indicated that DO and pH had a strong effect. Consequently, they determined the highest-ranking set of conditions from numerical optimization to maximize total cell numbers and phenotypic distribution (Table 1, Part B).

**TABLE 1:** Advances in microbioreactors for biomedical applications: patent trends and main design parameters.

Part A. Patent activity in microbioreactors for biomedical applications (2016–2026)										
ID/status	Product	Operation mode	Shear stress*	Microchannel cross-section geometry	Purpose	Sensor integration strategy	Cell retention	Input quantity	Output quantity	Year
WO2016044537A1 (Inactive)	Cells	Perfusion	Medium	Trapezoidal	A microfluidic system for high-throughput filtration and cell retention	None	Inertial filtration-based retention	2.5 × 10^6^ cells	1 × 10^8^ cells	2016
CN211159829U (Active	N/A	Batch	Low	Circular	Multi-channel bioreactor suitable for various reactions for timely detection	None	Not specified	N/A	N/A	2020
US11135589B2 (Active	Recombinant proteins	Perfusion	Medium	Trapezoidal	Effective control of the mammalian cellular growth to scale up the production of mAbs	None	Implied retention	2.8 × 10^6^ cells	1,120 pg of mAb/cell	2021
US20210252509A1 (Pending	N/A	Perfusion	Not specified	Rectangular	Switching from static cell seeding or producing cells to perfusion	None	Cell capture system with plurality of parallel pores smaller than circulating tumor cells	N/A	N/A	2021
US20230257689A1 (Pending	N/A	Batch/Fed-batch or continuous	Not specified	Circular Microliter plate format	Providing cell culture growth conditions through microposts that can be functionalized	Electrochemical: DO sensor	Functionalized microposts	N/A	N/A	2023

Part B. Main parameters in microbioreactor design according to application										
Product	Operation mode	Agitation (rpm)	Input quantity	Output quantity	Cultivation period (days)	Microchannel cross-section geometry	Sensor integration strategy	Cell type	Shear stress*	Cell retention	Ref
CD8 + central memory T cells	Perfusion	200	4 × 10^5^ cells/mL	9.01 × 10^6^ cells/mL	8	24 square bioreactors design based upon a 24-well plate Working volume per bioreactor well: 5 mL	Electrochemical: pH, temperature and DO sensors (pH:7.4; temperature: 37 °C; DO: 25%) pH control with CO_2_ and scheduled manual bolus addition of sodium bicarbonate buffer. DO control through O_2_ and N_2_ overlay	T cells	Medium	None	Amini et al. (2020)
CAR-Treg	semi-continuous perfusion	N/A	1 × 10^6^ cells/mL	1 × 10^7^ cells/mL	12	Rectangular	None	T cells	Low	Membrane-based	Edwards et al. (2026)
CAR T cells	Perfusion (automated)	Intermittent Mixing	2 × 10^6^ cells	>200 × 10^6^ cells	12	Irregular Growth chamber volume: 2 mL Microfluidic cassette dimensions (cm) Width (W): 5; Depth (D): 8; Height (H): 1	Optical: two optical density sensors Electrochemical:pH, DO, and temperature sensors (pH:7.4; DO: >80%) CO_2_ controller	Human primary T cells	Low	Membrane-based	Sin et al. (2024)
mAbs (transtuzumab)	Perfusion	120	30 × 10^6^ cells/mL	120 × 10^6^ cells/mL	17	Irregular Growth chamber volume: 2 mL Microfluidic cassette dimensions (cm) W: 5; D: 8; H: 1	Optical: two optical density sensors Electrochemical: two pH, DO, and temperature sensors (pH:7.4; DO: 40%) CO_2_ controller	CHO-K1	Low	Membrane-based	(Schwarz et al., 2023)
Cardioids	Periodic perfusion	N/A	300 cells per droplet	N/A	N/A	Irregular Microchannel dimensions (cm) W: 0.1; Length: 4; H: 0.5	None	mESCs	Low	Cell trap-based	Vertti-Quintero et al. (2025)
Retinal Organoids	Perfusion (RWV)	<30 (rotation)	1 NR/1 mL medium	N/A	25	Rotational spherical chamber	Electrochemical: (DO: 20% normoxia)	Mouse ESCs	Low	None	(DiStefano et al., 2018)
Cardiac Tissue (pcECM)	Perfusion	N/A	2.4 × 10^6^ cells/matrix	N/A	35	Scaffold-based (porous 3D)	Electrochemical (pH)	hMSCs	Low	Scaffold-based	(Markish et al., 2025)
Myotubes (aligned)	Static (Hydrostatic)	N/A	5–20 × 10^3^ cells/mm^2^	N/A	4	Rectangular W:300 µm	None	Primary myoblasts	Low	None	(Song et al., 2021)
Recombinant mAbs (anti-hIFN-α2b)	Perfusion	N/A	10^6^ cells/mL	N/A	18	Chambers H: 200 um	None	CHO-ahIFN-α2b	Low	None	(Bourguignon et al., 2022b)
hiPSC	syringe pump-driven perfusion	N/A	10^5^ cells/mL	N/A	5	Pillars H: 600 um	None	hiPSC (including fluorescent H2B-Cerulean line)	Low	None	(Bourguignon et al., 2022a)
Neural Organoids (Gyroid)	Static	N/A	9 × 10^3^ cells/well	N/A	77	Gyroid 3D architecture W: 200–800 µm	None	hiPSC-derived NES	Low	Scaffold-based	(van Altena et al., 2025)
Cardiospheres	Liquid Marble	N/A	3 × 10^5^ cells	3D organoids	-	Circular Diameter: 3.36 µm	Electrochemical (DO: 5%)	Cardiomyocytes	Not specified	Elastic shell with fine pores	(Aalders et al., 2021)
Hepatocyte metabolites	Perfusion	N/A	10^6^ cells/mL	Metabolic profile	5	Rectangular H: 300 μm; D: 50 µm	Electrochemical (pH:7.4; DO: 10%–15% *in vivo* mimic)	Rat Hepatocytes	Low	Scaffold slab-symmetrically fixed at the channel centerline	(Piemonte et al., 2018)
Retinal Organoids	Micro-millifluidic	N/A	1 organoid/chamber	2.5 mm organoid	30+	Rectangular channel H: 200 μm and chamber H: 2 mm	Electrochemical (pH:7.4; DO: 20%)	Human RtOgs (hiPSCs)	Low	Culture chamber	Xue et al. (2021)

Part C. Microbioreactor future perspectives										
Product	Operation mode	Agitation (rpm)	Input quantity	Output quantity	Cultivation period (days)	Microchannel cross-section geometry	Sensor integration strategy	Cell type	Cell retention	Ref
DNA/Bioparticles	Topological acoustofluidic	Acoustic vortices	200 nm particles	Controlled transport	N/A	N/A (PDMS microchamber with electroplated copper pillars)	N/A	N/A (Tracer particles)	N/A	(Zhao et al., 2025)
Neuro-proteins	Light-triggered release	N/A	10 mg Neuro-MOF	Controlled release	14 (healing)	Circular	Electrochemical pH sensor (pH:7.4)	Neuroblastoma/mast cells	Neuroblastoma membrane	(Zhao et al., 2023)

* Category assigned according to low shear stress ≤ 0.3 pa, medium shear stress 0.3–2.7 Pa and high shear stress ≥ 2.7 Pa (Patel et al., 2022) or low shear stress <1 Re, medium shear stress 1 to 50 Re and high shear stress 50–200 Re (White, 2009).

There is a growing field of evidence supporting the use of CAR regulatory T cells (Tregs) to regulate immune responses (Mohseni et al., 2020). To address this, microfluidic technology was integrated in biomanufacturing to significantly streamline the production of CAR-T cell therapies by increasing precision and control over fluid dynamics and cell manipulation.

A closed-system microbioreactor called the Mobius Breez microbioreactor (Erbi Biosystems, MilliporeSigma) was utilized to establish and assess a CAR T-cell production process. They achieved the production of over 60 million viable CAR T-cells derived from patient donors and more than 200 million viable CAR T-cells sourced from healthy donors, thereby satisfying the requisite minimum cell dosage for tisa-cel (Kymriah) and surpassing the maximum cell dosage for axi-cel (Yescarta), respectively (Sin et al., 2024). Notably, the cross-section of the growth chamber comprises a flexible gas-permeable polydimethylsiloxane (PDMS) silicone membrane whose cyclical deflection allows fast diffusion-based gas transfer and low-shear, bubble-free mixing of the growth chamber (Table 1, Part B).

Moreover, microfluidics offers the possibility of parallelizing multiple steps within the manufacturing workflow, enabling a more continuous and automated production line (Edwards et al., 2026). In this regard, researchers at King´s College of London (KCL) developed the KCL-Microbioreactor which is a closed microbioreactor that integrates the activation, transduction and expansion steps required for the manufacturing of functional CAR-Tregs from primary human Tregs with a 50% higher expansion capacity and a 92% increase in CAR-Treg yield, when compared to standard tissue culture plasticware. Furthermore, they reported a transduction efficiency of 67% due to an increase in cell-virus interactions (Edwards et al., 2026).

### 2.2 Recombinant protein production

Chinese hamster ovary (CHO) cells are the dominant platform for monoclonal antibody (mAb) production, and microscale technologies increasingly support upstream process development. In CHO cell systems, microfluidic bioreactors are primarily applied to process optimization and analytical enhancement rather than developmental control. Due to the high complexity of media for mammalian cell culture, the strategy of one-factor-at-a-time has been considered ineffective. Therefore, the statistical approach of design of experiment (DoE) has been adopted to reduce the number of experiments (Ritacco et al., 2018; Schwarz et al. (2023) enhanced monoclonal antibody (mAb) production in CHO cell lines by applying a Design of Experiments (DoE) strategy for medium optimization (Schwarz et al., 2023). This approach allowed them to tailor key nutrient concentrations in the perfusion medium and establish a targeted cell-specific uptake rate. By utilizing the Erbi Breez perfusion microbioreactor, the authors significantly minimized experimental effort while evaluating critical process parameters, including temperature, pH, dissolved oxygen (DO), and perfusion rate. Ultimately, their work underscores the critical role of precise parameter regulation during steady-state perfusion operations, as even minor disturbances can lead to significant deviations from the steady state (Table 1, Part B); (Schwarz et al., 2023).

In the realm of mAb productivity increment, Bourguignon et al. (2022a) utilized a cistern-based microfluidic device to achieve high-density cultivation of CHO-ahIFN-α2b cells, enabling continuous recombinantmAb production over 18 days. By maintaining a stable microenvironment through laminar flow and 24-h perfusion cycles, the platform reached a mAb productivity nearly six times higher than traditional T-flask methods. Furthermore, the antibodies produced exhibited superior biological performance, including twice the binding affinity and significantly enhanced cytokine neutralizing activity compared to standard controls. This approach demonstrates a cost-effective, scalable alternative for manufacturing high-quality biopharmaceuticals through precise microenvironmental control (Bourguignon et al., 2022b).

In terms of antibody production at-line monitoring, Pinto et al. (2021) developed a multiplexed microfluidic cartridge for antibody production in CHO cultures. The miniaturized immunoassay platform enabled rapid protein quantification with minimal sample volume, supporting real-time productivity assessment. Integration of such analytical modules aligns with Quality by Design frameworks by enabling tighter process monitoring and feedback control (Pinto et al., 2021).

#### 2.2.1 Bridging process intensification, perfusion strategies, and high-density cultivation

Perfusion culture maintains high viable cell densities while continuously harvesting products and removing metabolic waste. From a design perspective, ensuring uniform operation across parallelized microfluidic units requires careful control of hydrodynamic resistance and flow distribution (Schimek et al., 2018). Small variations in channel geometry or surface fouling can introduce significant deviations in shear stress and nutrient delivery. To mitigate this, microfluidic architectures increasingly incorporate flow equalization strategies, including pressure-balanced channel networks, integrated flow sensors, and automated feedback control systems. These design considerations are critical when transitioning from laboratory-scale devices to scalable platforms, where maintaining consistent microenvironmental conditions across multiple units becomes essential for reproducibility and process robustness (Marques and Szita, 2017). Indeed, patent activity highlights cell growth under these conditions and also advances in cell retention (Table 1, Part A).

In this regard, perfusion microbioreactors provide innovative solutions for cell retention without conventional membrane filtration. Schellenberg et al. (2023) implemented a 3D-printed microfluidic spiral separator exploiting inertial forces to achieve CHO cells label-free separation from supernatant. This configuration enabled continuous product recovery while minimizing fouling and shear-induced damage. Such microfluidic separation modules can function as intensified unit operations within perfusion workflows, reducing reliance on traditional tangential flow filtration systems (Schellenberg et al., 2023).

Although CHO cells tolerate higher shear stress than pluripotent stem cells, excessive hydrodynamic forces can negatively impact viability and therapeutic antibodies glycosylation profiles (Li, et al., 2010). Then, precise shear exposure modulation is achieved through microfluidic systems as they operate under predictable laminar flow conditions.

Regards oxygen consumption, high-density cultures exceeding 10^7^ cells·mL^-1^ demand robust oxygen transfer and metabolic regulation. Therefore, microfluidic systems fabricated from gas-permeable materials or incorporating dedicated oxygenation channels can stabilize DO concentrations at microscale volumes. While volumetric oxygen transfer coefficients may be lower than in stirred-tank reactors, short diffusion distances and high surface-to-volume ratios partially compensate in small-scale applications.

This technical management of the microenvironment establishes a robust framework for refining the theoretical principles of bioreactor engineering, as showcased by Piemonte et al. (2018); (Piemonte et al., 2018). In their research, an advection–diffusion–reaction transport model was employed to fine-tune 3D hepatocyte cultures within a biomimetic, sinusoid-inspired geometry that accurately simulates the endothelial–parenchymal interface. By evaluating the Péclet and Thiele dimensionless numbers, the authors offered a clear scientific rationale for how oxygen and nutrient delivery are maximized within micro-architectures that mirror hepatic sinusoids. This approach yielded a standardized, high-performance platform capable of sustaining high-density cell populations and modeling conditions like non-alcoholic fatty liver disease (NAFLD), ultimately surpassing conventional static culture techniques in simulating *in vivo* liver metabolism and mass transport.

### 2.3 Engineering the 3D hiPSCs niche for expansion and differentiation

#### 2.3.1 Harnessing hydrodynamics and shear stress management

HiPSCs exhibit marked sensitivity to mechanical stress, particularly during single-cell passaging or early aggregation stages. Shear stresses above ∼1–2 dyn cm^-2^ can induce apoptosis, disrupt colony morphology, and compromise pluripotency marker expression (Kehoe, et al., 2010). Consequently, microfluidic bioreactor design for hiPSCs prioritizes laminar flow regimes and geometrical features that minimize wall shear stress while preserving convective nutrient delivery.

Microchamber expansions, perfusion-through-hydrogel architectures, and low-flow perfusion systems are commonly employed to decouple mass transport from mechanical load. Marsano et al. (2016) demonstrated a perfused microfluidic heart-on-chip system in which hiPSC-derived cardiomyocytes were embedded within a 3D construct exposed to controlled perfusion and cyclic mechanical stimulation. Importantly, the platform enhanced sarcomeric organization and contractile maturation without inducing detrimental stress responses. These findings underscore that controlled hydrodynamics can support lineage maturation when shear profiles are tightly regulated (Marsano et al., 2016).

#### 2.3.2 Mass transport and oxygenation in 3D hiPSC-Derived constructs

Diffusion limitations are a major bottleneck in static 3D cultures of hiPSC aggregates and organoids. In fact, oxygen and nutrient gradients become critical beyond 300–500 μm, frequently resulting in hypoxic cores and necrotic regions. Notably, microbioreactors overcome this limitation by introducing controlled convective transport while maintaining microscale precision (McMurtrey, 2016).

In the context of mass transport (Cho et al., 2021), developed a microfluidic platform integrating extracellular matrix (ECM) components with periodic medium flow to culture brain organoids derived from hiPSCs. The perfused microenvironment improved structural organization and functional neuronal maturation relative to static conditions. Beyond simple oxygen delivery, convective flow modulated ECM remodeling and mechanotransduction pathways, suggesting that hydrodynamics influence developmental signaling cascades. These observations reinforce the concept that microfluidic perfusion is not merely a transport mechanism but an active regulator of tissue morphogenesis.

Bourguignon et al. (2022b) demonstrated this approach by developing a microfluidic millibioreactor that incorporates an optimized internal architecture of microscale pillars (micropillars). This multiscale design enables homogeneous flow distribution and stable perfusion, thereby improving nutrient transport while minimizing shear stress. Their system successfully maintained hiPSC viability and pluripotency under continuous flow conditions, highlighting the critical role of chamber geometry and controlled perfusion in overcoming diffusion-related limitations in larger fluidic volumes (Bourguignon et al., 2022a).

#### 2.3.3 Spatiotemporal control of differentiation signals

Conventional bulk systems are limited by dead volume and slow media exchange kinetics hampering directed differentiation protocols which often require sequential activation and inhibition of signaling pathways, such as canonical Wnt modulation in cardiomyocyte induction. In contrast, microbioreactors allow rapid media formulation switching and precise gradient formation (Zhang, et al., 2017).

The microchannel small internal volumes enable dynamic morphogen exposure with high temporal resolution, facilitating reproducible lineage commitment. Furthermore, gradient-generating microarchitectures permit spatial patterning of differentiation cues, enabling systematic analysis of morphogen concentration thresholds (Sloan, et al., 2020). Such capabilities are particularly advantageous in disease modeling and developmental biology applications where subtle temporal differences in signaling influence fate outcomes.

### 2.4 Organoid microbioreactors

Organoids are 3D multicellular structures derived from stem cells or primary tissue-derived cells that recapitulate key aspects of native tissue architecture and function, including cellular diversity, spatial organization, and physiological behavior resembling those of *in vivo* tissues and organs (Vertti-Quintero et al., 2025). It has been reported that the culture of pluripotent stem cells within confined nano/microliter volumes enables a more rapid accumulation of secreted molecules around cells, regulating their processes of self-renewal and differentiation trajectories (Guild et al., 2016). In addition, by providing controlled medium perfusion at the micrometer scale, microfluidic devices allow a tight balance between the addition of exogenous molecules and the accumulation of autocrine cues to direct cell differentiation while maintaining their capacity for cell self-regulation (Luni et al., 2022).In this realm (Vertti-Quintero et al., 2025), demonstrated that 7 µL microdroplets are sufficient to expand and differentiate mouse embryonic stem cells (mESCs) at various developmental stages.

Building on the need for standardized tissue production, Xue et al. (2021) validated an automated micro-millifluidic platform that generates high-quality human retinal organoids with mature photoreceptor morphologies (Xue et al., 2021). This nearly labor-free system optimizes metabolic states through shear-free perfusion, allowing for non-invasive monitoring and long-term maintenance exceeding 30 days. Such precise environmental control is essential for ensuring consistency in replacement therapies and drug screening applications. In a continued effort, DiStefano et al. (2018) demonstrated this by utilizing Rotating-Wall Vessel bioreactors to accelerate the maturation of mouse retinal organoids. The system’s superior mass transfer and low shear stress produced organoids 140% larger than static controls, achieving postnatal-like synaptogenesis by Day 22 and providing a high-speed platform for disease modeling (DiStefano et al., 2018) (Table 1. Part B).

On the other hand, the impact of microfluidic topological constraints on the development of functional tissues has been explored. Song et al. (2021)investigated how microchannel design influences the alignment and maturation of primary myoblast-derived myotubes over a 4-day differentiation period. The researchers demonstrated that while both solid and dashed walls successfully induced cell alignment mimicking native skeletal muscle, the dashed constraints significantly facilitated maturation by creating low-velocity zones that optimized cell seeding uniformity. Therefore, myotubes cultured under these topological constraints showed increased cell area and a higher nuclei per myotube density compared to those in continuous wall architectures. This specialized microfluidic platform, which improves adhesion between PDMS and polystyrene, provides a robust, biomimetic model for the *in vitro* study of skeletal muscle physiology and metabolism (Song et al., 2021).

Within this framework, the study developed by Markish et al. (2025) is essential for discussing how perfusion bioreactors can move beyond simple cell expansion, enabling the complex tissue maturation through a modular perfusion bioreactor that provides synchronized, physiological-like electrical and mechanical stimuli to bone marrow primary human mesenchymal stem/stromal cells on thick porcine cardiac ECM (pcECM) scaffolds. By mimicking left ventricle inflation and action potential profiles over 35 days, the system successfully induced cardiac progenitor markers and dense tissue formation. This biomimetic approach significantly improves cellularization and proliferation, offering a robust platform for engineering functional living cardiac tissue (Markish et al., 2025).

Beyond fluid dynamics, the physical architecture of the growth environment is critical for tissue-mimetic engineering. van Altena et al. (2025) addressed this by developing a high-resolution micro-digital light processing protocol to fabricate 3D hydrogel scaffolds with gyroid-based architectures (van Altena et al., 2025). Using a biocompatible Polyethylene Glycol Diacrylate (PEGDA) and gelatin methacryloyl (GelMA) PEGDAGelMA with feature sizes down to 12.4 µm, these meso-scale structures—including hollow variants for hosting pre-formed organoids—promote strong cell adhesion and sprouting. Collectively, these studies illustrate how the integration of advanced 3D fabrication and dynamic perfusion overcomes the physiological bottlenecks of traditional culture methods.

#### 2.4.1 Scaffold-free 3D cultures

Besides the diffusion and maturation constraints inherent in static 3D systems, researchers are developing dynamic, scaffold-free environments that prioritize microscale precision and enhance mass transfer. Aalders et al. (2021) addressed this by utilizing superhydrophobic liquid marble microbioreactors, which create a non-adhesive confined space that stimulates rapid cell coalescence and ensures optimal gas exchange. This protocol facilitates highly reproducible 3D cocultures of cardiomyocytes and fibroblasts, enabling the study of paracrine signaling and disease modeling within just 24 h (Aalders et al., 2021). Ultimately, these scaffold-free microenvironments provide a physiologically relevant architecture that significantly outperforms traditional monolayer models for high-throughput drug screening (Table 1. Part B).

Similarly, droplet-based microfluidic systems have emerged as highly controllable scaffold-free platforms for organoid and spheroid generation. Sart et al. (2022) highlighted how droplet microfluidics enables reproducible aggregate formation, controlled nutrient diffusion, and high-throughput manipulation of multicellular systems while preserving physiologically relevant cell–cell interactions (Sart et al., 2022). Likewise, hanging-drop microfluidic approaches facilitate uniform spheroid formation under dynamic perfusion conditions, improving reproducibility and mass transport compared to static cultures (Huang, et al., 2020).

### 2.5 Emerging technologies/future perspective

Perspective explores the integration of advanced physics and bio-interfacial engineering to push the microscale control boundaries. The field is moving toward responsive, “intelligent” systems, as demonstrated by Zhao et al. (2023), who developed a neuro-inspired biomimetic microreactor (Neuro-MOF) (Zhao et al., 2023). By camouflaging metal-organic frameworks with neuroblastoma membranes, this system achieves near-infrared light-triggered protein release to initiate peripheral nerve regeneration and hair follicle neogenesis. This approach not only suppresses inflammation but also promotes M2 macrophage polarization, restoring sensory function in deep skin burn models within 14 days and showcasing the potential for precisely controlled therapeutic delivery in complex wound management. In parallel, new physical paradigms are redefining particle manipulation. Zhao et al. (2025) introduced on-chip topological acoustofluidic devices that harness elastic valley spin and nonlinear fluid dynamics to create “topological pressure wells” (Zhao et al., 2025). This technology functions as a high-precision molecular tweezer, trapping DNA and 200 nm particles with energy intensities two orders of magnitude higher than conventional devices. By utilizing energy-efficient edge modes for backward-immune transport, this platform bridges topological physics and life sciences, offering a standardized tool for the deep-subwavelength manipulation of nanomaterials. Together, these innovations signal a shift toward microreactors that are not only faster and more efficient but also capable of active, real-time interaction with biological systems (Table 1. Part C).

## 3 Discussion

Traditional large-volume bioreactors are designed to support cell growth through controlled nutrient delivery and gas exchange, albeit are poorly suited to the decentralized, small-batch production required for personalized therapies such as CAR T-cells (Edwards et al., 2026; Jagannathan et al., 2026).

Microbioreactor design has emerged as a versatile engineering framework capable of addressing distinct biological objectives across eukaryotic cell systems. The microbioreactor integration into CAR T-cell manufacturing represents a significant advancement in achieving numerical optimization for both total cell count and phenotypic distribution. In this setting, Jagannathan et al. (2026) developed a modeling framework designed to estimate, in real time, the individual metabolic rates of T cells cultured *ex vivo* within a microbioreactor (Jagannathan et al., 2026). On top of this, by parallelizing critical steps—such as activation, transduction, and expansion—into a continuous, automated, and closed workflow, these systems drastically improve production efficiency. A prime example is the KCL-Microbioreactor, which leverages enhanced cell-virus interactions to reach a 67% transduction efficiency, outperforming standard tissue culture plasticware with a 50% higher expansion capacity and a 92% increase in CAR-Treg yield. These outputs satisfy the minimum dosage requirements for tisa-cel (Kymriah) and surpass the maximum dosage thresholds for axi-cel (Yescarta).

In the context of hiPSCs, the principal value of microfluidics lies in its capacity to precisely regulate shear stress, oxygenation, and morphogen dynamics while enabling controlled 3D tissue organization. By decoupling convective transport from mechanical overload and facilitating rapid temporal modulation of signaling pathways, these platforms enhance reproducibility and functional maturation of stem cell–derived constructs.

In terms of reproducibility, Seiler et al. (2022) reported a modular automated microfluidic culture platform enabling multiplexed experimentation with integrated flow control. For hiPSC systems, automation reduces variability in feeding schedules, oxygen tension, and differentiation timing while lowering reagent consumption (Seiler et al., 2022).

Despite these advantages, scale-up remains challenging. Microfluidic systems predominantly rely on scale-out strategies through parallelization (Hwang et al., 2025). While this approach enhances environmental uniformity and reproducibility, integration into Good Manufacturing Practice (GMP)-compliant workflows requires careful engineering validation. Nonetheless, for applications emphasizing phenotypic fidelity and tissue maturation, microbioreactors provide unmatched control over the stem cell microenvironment. This feature positions microbioreactors as powerful tools for cell-based screening studies (Frey and Krull, 2020), autologous cell therapy (Sin et al., 2024), organoid development (Vertti-Quintero et al., 2025), disease modeling (Piemonte et al., 2018), and regenerative medicine workflows (Liu et al., 2013).

Critically, maintaining environmental uniformity across parallelized microfluidic units remains a non-trivial engineering challenge. Variations in flow resistance, channel fouling, or minor fabrication inconsistencies can lead to heterogeneous shear profiles and nutrient gradients across units. To address this, advanced system-level design strategies incorporating real-time feedback control, integrated flow sensors, and automated pressure regulation are required. Furthermore, the transition toward GMP-compliant manufacturing demands closed-loop architectures capable of continuous monitoring of critical quality attributes (CQAs), including DO, pH, and metabolite concentrations. In this context, microfluidic platforms must evolve from isolated experimental devices into fully integrated bioprocess systems, where automation ensures both scalability and regulatory traceability.

Conversely, in CHO and other mammalian production cell lines, microbioreactors primarily contribute to process intensification and analytical integration rather than microenvironmental biomimicry. Their strengths reside in high-resolution process monitoring, efficient cell retention strategies for perfusion culture, and reduced reagent consumption during clone screening and media optimization. While volumetric limitations currently preclude their widespread adoption as standalone manufacturing platforms, microfluidic modules significantly enhance upstream development and enable data-rich optimization under quality by design paradigms. Collectively, these complementary applications illustrate how microbioreactor technologies can bridge fundamental cell biology and translational bioprocess engineering within biomedical contexts.

In the realm of material selection, it critically influences microfluidic performance. While PDMS offers optical transparency and gas permeability, its absorption of hydrophobic small molecules can distort effective concentrations of differentiation factors. Other drawbacks are the swelling in organic solvents, the inherent hydrophobicity and the potential water evaporation through the material (Matining et al., 2025). To address this challenge, thermoplastics such as: poly (methyl methacrylate) (PMMA), cyclic olefin polymer (COP), cyclic olefin copolymer (COC) are attractive in microfluidic device fabrication due to their cost-effectiveness, good biocompatibility, gas permeability, low electrical conductivity, organic solvent resistivity and good optical properties (Shakeri et al., 2022). In contrast, the main limitations of using thermoplastic polymers are associated with thermal deformation, weak gas permeability, possible autofluorescence, difficult direct or indirect bonding and sensor integration (Zimina et al., 2024). However, thermoplastics are typically used in injection molding and hot embossing. The former is employed for high throughput, and cost-efficient microfluidic device production (Gale et al., 2018). In the same vein, material selection is particularly critical in mAb production due to potential protein adsorption as PDMS can sequester hydrophobic molecules and adsorb therapeutic antibodies, leading to quantification artifacts and product loss. Consequently, thermoplastics and chemically resistant polymers are increasingly preferred for production-oriented microfluidic systems.

Another alternative are 3D-printed resins which are increasingly being adopted. Khan et al. (2021) demonstrated a 3D-printed microbioreactor compatible with organoid culture and live imaging, illustrating the feasibility of rapid prototyping combined with stem cell compatibility (Khan et al., 2021).

Indeed, bioinks, defined as printable biomaterial formulations containing cells and biocompatible polymers, have been developed to 3D print structures in heatable resin vats using commercial micro digital light processing (µDLP) printers. μ DLP employs a layer-by-layer approach (Amini et al., 2023) that can achieve a feature resolution of approximately 5–10 μ m with acrylate polymers. This resolution has been explored in the presence of soft hydrogels (van Altena et al., 2025). These researchers developed a PEGDA/GelMA-based bioink to print complex, highly porous, millimetre-sized, gyroid-based 3D hydrogel scaffolds for neural cell/organoid culture that showed that this material enhances more homogeneous cell colonization compared to PEGDA-only scaffolds. In particular, these bioinks overcome the thermoplastic gas permeability limitation and also exhibited minimal autofluorescence, facilitating high-resolution confocal immunofluorescence imaging.

Despite promising advances, volumetric throughput constraints limit the deployment of microfluidics as primary manufacturing bioreactors. Instead, their principal impact lies in clone screening, media optimization, perfusion module development, and real-time analytical integration. Scale-out approaches may support modular manufacturing concepts. For instance, parallelized microfluidic platforms that successfully incorporate 256 staggered herringbone micromixers to ensure industrial scale manufacturing of RNA-loaded lipid nanoparticles (Hwang et al., 2025). However, regulatory validation across parallelized units remains a practical consideration.

In the context of organoids, droplet microfluidics offers a suitable platform for scalable and time-resolved 3D culture (Sart et al., 2022). Moreover, it has the potential to modulate cell differentiation by enhancing direct cellular contact and regulating the molecular transport of nutrients and secreted morphogenes. These features enable the recreation of precise environmental niches for *in vitro* tissue development (Vertti-Quintero et al., 2025).

Overall, mammalian cell production microbioreactor lines emphasize process intensification, analytical precision, and modular integration. Within the broader context of biomedical applications, these systems complement traditional bioreactors by accelerating development cycles and enhancing process understanding rather than replacing large-scale production infrastructure.

Advanced microfluidic bioreactors redefine bioprocessing by integrating precise dimensionless transport control with complex 3D architectures, achieving mAb productivities up to 6x higher than static methods. Platforms utilizing advection–diffusion–reaction models optimize nutrient delivery through specific Péclet/Thiele regimes, maintaining high-density viability and accelerating maturation, such as producing retinal organoids 140% larger with synaptogenesis by Day 22 (Bourguignon et al., 2022b; DiStefano et al., 2018; Piemonte et al., 2018).

Topological engineering, including 3D gyroids with 12.4 µm features and dashed-wall constraints, further enhances tissue-mimetic performance by optimizing cell seeding and alignment. Emerging “intelligent” paradigms, such as NIR-triggered Neuro-MOFs for nerve regeneration and topological acoustofluidics with 100x higher energy intensity, enable active, real-time biological interaction. This convergence of microscale precision and dynamic perfusion shifts the field toward standardized, high-fidelity translational medicine (Song et al., 2021; Van Altena et al., 2025; Zhao et al., 2023; 2025).

Ultimately, collaboration between academia and industry is necessary to promote the establishment of unified standards that will enhance the reproducibility, scalability, and commercialization of microfluidic technologies. In fact, microfluidic bioreactors should not be viewed as direct substitutes for conventional systems, but rather as enabling technologies that bridge microscale biological precision with macroscale manufacturing requirements. Their true impact lies in redefining how cellular microenvironments are engineered, measured, and controlled, thereby accelerating the convergence between cell biology, bioengineering, and translational medicine. By drastically reducing reagent requirements and accelerating biological maturation, microfluidic bioreactors contribute to mammalian cell products obtention and precision medicine model creation for drug screening to minimize unnecessary toxicity for patients. Consequently, it transforms high-end science into a tool for global health accessibility.
